# Lip print enhancement: review

**DOI:** 10.1080/20961790.2020.1751396

**Published:** 2020-05-05

**Authors:** Maxwell Abedi, Constance Afoakwah, Dan Nana Osei Mensah Bonsu

**Affiliations:** a Institute of Forensic Science, Gujarat Forensic Sciences University, Gandhinagar, Gujarat, India; b Department of Forensic Sciences, University of Cape Coast, Cape Coast, Ghana

**Keywords:** Forensic sciences, cheiloscopy, lip print, lysochrome, fluorescent dye, persistent lipstick

## Abstract

Lip print (LP) evidence can be an essential tool for human forensics. LPs have conventionally been developed using substances such as lysochrome dyes, fluorescent dyes, indigo dye, aluminium powder, and silver metallic powder. However, techniques for LP enhancement from various substrates are currently inconsistent and lack standardisation in practice. This review summarises current knowledge on the physical and chemical techniques of LP enhancement, identifies limitations, and provides suggestions for future research on practical applications of cheiloscopy as a forensic tool in criminal justice.

**Key points**
- The grooves and wrinkles of the human lip establish unique patterns that persist throughout life.
- Cheiloscopic patterns exhibit discriminatory individual characteristics that may constitute circumstantial forensic evidence.
- Enhancement techniques for latent lip prints on porous and nonporous substrates can be classified as physical or chemical.
- Unlike fingerprint, there is a current lack of consistency and/or standardisation on latent lip print enhancement methods in frontline forensic practice.

## Introduction

Latent forensic mark evidence of fingerprints, palm prints, and often even footprints can be decisive in resolving criminal cases. Whereas enhancements for the foregoing evidence types are well-known, there are minimal data on the impression produced by the human lip. A lip print (LP; *Figura linearum labiorum robrorum*) forms when the characteristic patterns of the grooves and wrinkles (*Sulci labiorum roborum*) of the labial mucosa come into contact with a surface [1,2]. The study and evaluation of LPs (i.e., lip marks), based on the assumed concept of labial grooves’ uniqueness, is termed cheiloscopy or queiloscopy [3].

The vermillion border of the lips has sweat and sebaceous glands. These glands’ secretions (together with the moisturising activity of saliva from the tongue), upon contact with a surface, form the basis of LPs. The presence of LPs may constitute circumstantial evidence [4]. The grooves and wrinkles on lips begin development at approximately 6 weeks estimated gestational age and once established, these unique patterns [5] persist throughout life except after a deformity (through injury or disease) or putrefaction [6,7].

The increasing sophistication of crime, and the tendency of criminals to adopt trail-covering precautionary measures during the commission of a crime [8], have led to advocacy for the use of lip marks as additional tools in forensic investigations of crime [2]. Furthermore, the pattern of labial wrinkles and grooves exhibits discriminatory individual characteristics, with a scope that has been equated to fingerprints [9]. Cheiloscopy may thus prove valuable in criminal profiling [10]. Neo et al. [11] showed that proper LP analysis could be useful in estimating gender, race, crime type, as well as the number of persons involved in a crime [11]. Fonseca et al. [12] reported a useful review of real cases that utilise cheiloscopy. LPs are common on surfaces such as clothes, drinking cups and glasses, cigarette butts, envelopes, and cutlery items, and may be visible (patent) or invisible (latent). Latent LPs are common because of increased use of so-called transfer-resistant or permanent lipsticks, and the fact that most males generally wear no lipsticks at all [13]. Enhancement of latent LPs relies on the reaction of sweat and sebum constituents deposited on surfaces with chemical agents [6].

Effective LP analysis, as a forensic investigative tool, requires a continued refinement of existing methods and developing new robust techniques. This paper reviews the enhancement techniques employed in cheiloscopy, identifies challenges and gaps in existing methods and provides a perspective on future research.

## Methods

We searched two electronic databases, Scopus and Web of Science, for peer-reviewed articles that are relevant to LP enhancement techniques. Specifically, we searched for work with the keywords lip print*, cheiloscopy*, latent*, or lip print* in any field; this produced 127 results (January 2000 – August 2019). After a review of titles and abstracts, we selected 16 original articles (out of the 50 articles directly related to forensic science) that clearly included techniques and/or methods for LP enhancement. We also reviewed reference lists within the identified forensic publications to obtain additional sources. We repeated this search in the PubMed and Google Scholar databases as additional checks on the completeness of our search strategy.

## Results and discussion

### LP enhancement

Researchers’ initial approach to LP analysis was based on the traditional methods of developing fingermarks. Efficient development of latent LPs, however, requires a rigorous review of conventional reagents used for fingermark enhancement that make use of differences in their constituents [13]. The techniques used for enhancing LPs from various porous and nonporous surfaces can be classified as physical or chemical. Table 1 summarises the various physical and chemical enhancement methods for LPs.

**Table 1.** Physical and chemical enhancement methods for LPs.

Reference	Design details	Results
Seguí et al. (2000), [13]	Persistent lipstick was applied on the lips of volunteers, and then pressed on ceramic, glass, cotton fabric, and white paper substrates. LPs on each substrate were enhanced with developers (aluminium powder, cobalt oxide powder, and magnetic powder) after exposure to ambient conditions.	Identifiable LPs were obtained up to 30 days after deposition. Aluminium powder and magnetic powder gave better enhancement efficiency than magnetic powder.
Dolly et al. (2016), [18]	Visible and latent LPs were produced on various supports (white cotton fabric, white satin fabric, and white clay cup) from 60 individuals and developed using Sudan III dye, indigo blue dye, and aluminium powder.	Sudan III dye and aluminium powder showed significant enhancement of visible LP. Indigo dye is useful for developing both visible and latent LPs.
Khanna et al. (2010), [3]	Traditional and long-lasting lipsticks were used to obtain visible and latent LPs, respectively, from 45 volunteers on bone China cup, white satin fabric, and white cotton fabric. Samples were developed by dusting with a camel hair brush and were lifted using an appropriate adhesive tape.	There was no appreciable difference between the enhancement efficiency by natural (vermilion and indigo dye) and lysochrome (Sudan Black) dyes.
Fonseca et al. (2014), [19]	Latent LPs were produced on metallic straw (*bombilla*). Volcano, fluorescent, and Silk Black volcano powders were utilised for print enhancement.	White volcano powder was most effective for LP development, followed by fluorescent powder and Silk Black volcano powder.
Castello et al. (2002), [27]	Lipstick was applied on the lips of 10 volunteers. Prints were produced and developed on tissue paper and white cotton fabric using lysochrome dye (Sudan III, Sudan Black, and Oil Red O), ninhydrin, and fingerprint (FP) powders (FP red powder, FP black powder, and silver metallic powder).	There was a quality enhancement of prints on both substrates using solution or powder lysochrome dyes (in the order: Sudan Black > Oil Red O > Sudan III). Ninhydrin produced a negative result.
Navarro et al. (2006), [29]	A total of 17 corpses were utilised. A mould made of silicone was created to simulate the human lip. A protective or long-lasting lipstick was spread on the mould and afterwards pressed against the corpse’s skin (right side of the neck and the anterior region of the forearm). Development of LP was initialised with powder lysochromes (Sudan III, Sudan Black, and Oil Red O).	Sudan Black produced better results than Sudan III and Oil Red O in terms of enhancing latent LPs.
Kumar et al. (2010), [4]	Latent LPs of 200 participants were made on a rough surface without applying any lipstick. Prior to print production, each lip was cleaned with gauze moistened in saline water and dried with sterile cotton. Lysochrome dye (Oil Red O) and fluorescent dye (Nile Blue) were utilised for latent LP enhancement.	There were clear and well-defined lip grooves and wrinkles in prints developed with fluorescent dye relative to those enhanced with lysochrome dyes.
Castelló et al. (2005), [30]	The study utilised existing pre-existing latent LP (over 1 year old) produced on a colored substrate. Nile Red (fluorescent dye), both in powder and as an alcoholic solution, were used to develop prints. UV light (320–400 nm) and an alternate light source (390–520 nm) were utilised for print visualisation.	Good-quality prints (with well-defined shapes and outlines) were obtained with fluorescent dye (Nile Red).

### Physical methods

Physical methods encompass enhancement and visualisation of latent LPs with the aid of finely divided powder particles that physically adhere to the aqueous (moisture) and sebum components in the latent print residue on surfaces [14]. The powders consist of a resinous polymer (starch, kaolin, rosin, and silica gel) for adhesion and pigments (antimony trisulfide, lead iodide, or mercury sulphide) for contrast [15]. The physical methods for latent LP enhancement are akin to those employed in developing and visualising latent fingermarks. Various researchers have documented the use of aluminium powder, silver metallic powder, silver nitrate powder, plumb carbonate powder, fat black aniline dye, or cobalt oxide and other paraphernalia in enhancement and visualisation of latent LPs [13,16,17]. The development process entails dusting the latent print with powder using magnet (in the case of magnetic powder) or a bristle brush, and lifting the print using an appropriate adhesive tape [13].

Seguí et al. [13] studied the efficacy of aluminium, magnetic, and cobalt oxide powder in enhancing latent LPs from nonporous surfaces (glass and ceramics), and obtained identifiable prints after 30 days. Similarly, Dolly et al. [18] obtained significant print development using Sudan III dye and aluminium powder. Furthermore, Khanna et al. [3] asserted that natural dyes (vermilion and indigo dye) have an enhancement efficiency akin to that of lysochrome dye (Sudan Black) in the enhancement of latent and visible prints from bone China cup, white satin fabric, and white cotton fabric. The resulting LP quality was less efficient on cotton fabric compared with that on cup and satin fabric, because of the higher absorbance of lipstick components by the cotton fabric. To ascertain the prospects for enhancement on atypical surfaces, Fonseca et al. [19] proposed an experimental model that employed metallic straw (*bombilla*) as a substrate onto which LP was deposited. White volcano powder was the most effective in LP development, followed by fluorescent powder and Silk Black volcano powder. However, conventional powder techniques are generally not suitable for processing LPs, because the brushes used tend to smear and leave streak marks that may falsely contribute to individual characterisation. Sudan dyes are also category 3 carcinogens that may present human health risks [20,21].

Kapoor and Badiye [22] employed still digital photography to profile the LP patterns in an Indian population based on the Suzuki and Tsuchihashi classification pattern [23,24]. Although this approach is relatively simple and eliminates the laborious steps in traditional methods, it does not apply to LP samples (which still require enhancement). Nevertheless, this approach is suitable for probable, noninvasive exemplar collection for suspects.

### Chemical methods

Chemical techniques employed for LP enhancement relies on the biochemical reaction of lipid residues (deposited on surfaces) with chemical agents [6]. The most utilised compounds that have lipophilic properties for LP development include lysochrome [17,25] and fluorescent dyes (Nile red) [26]. Lysochrome is a generic term for chemical agents that can stain fatty acids *via* one portion (lyso) dissolving upon contact with fat, and the other (chrome) producing a colour to reveal the print patterns [25].

Castello et al. [27] studied the effectiveness of three lysochrome compounds (Sudan III, Sudan Black, and Oil Red O) in developing new and old LPs from long-lasting lipsticks on tissue paper and cotton fabric, compared with ninhydrin and other fingerprint powders. The LP quality improved on both substrates using solution or powder lysochrome dyes (in the order: Sudan Black > Oil Red O > Sudan III), although pattern quality was substrate-dependent and reduced in accordance with increasing LP age. Ninhydrin produced a negative result because of the absence of amino acids in the LP residues, a precursor for its reaction [28]. In a related work exploring the efficacy of lysochrome dyes in the development of invisible lipstick-contaminated LPs on human skin, Sudan III dye, Oil Red O, and Sudan Black were efficient for obtaining recent LPs on a corpse’s skin [29].

Fluorescent dyes perform better [26] at enhancing LPs on coloured or multicoloured surfaces than lysochrome dyes because of lysochrome dyes’ contrast problems [30]. In a comparative study to evaluate the potency of the aforementioned dyes in LP enhancement, lip grooves and wrinkles were sharper and well-defined in LPs developed with fluorescent dye than with lysochrome dyes [4]. This result is consistent with previous work [17,18,25,29,30]. Natural dyes (vermilion and indigo dye) exhibit the same enhancement efficiency as the lysochrome dye Sudan Black [3] on LPs from long-lasting lipstick on different substrates (cup, static fabric, and cotton fabric).

## Future directions

Available data on LP enhancement methods indicate use of laboratory simulations (mock cases) with conditions that favoured print development. The outcomes of such procedures, albeit useful as proofs of concept, are not entirely applicable and/or replicable in actual casework scenarios in which other matrices may interfere with LP enhancement. Furthermore, data from most studies have focused on enhancement of prints made through prior application of visible lipsticks, instead of those from persistent lipsticks or individuals (mostly males) who rarely wear lipstick. Such approaches, which are mostly undertaken for convenience, render extrapolation of results problematic given that the samples used are not representative of the population. The effect of various environmental conditions such as temperature and humidity on LP persistence on different substrates, and the concomitant influences on print development, including technique choice, is currently unknown.

The quality of impression-related evidence is considerably influenced by the pressure applied at the time of deposition. However, the effect of the applied pressure during LP deposition, the consistency of the print patterns (given that it is produced by the mobile portion of the lip) in relationship to the deposition pressure, and the impact on enhancement remain unexplored. Unlike fingerprints, there are currently no standard techniques for LP development. The absence of method validation and quality assurance in this area has inadvertently contributed to the limited acceptance of LP evidence in the justice system despite the probative potential for an improved justice outcome. Hence, future research should be targeted towards addressing these enumerated limitations, to introduce a degree of consistency in using and applying LP enhancement techniques.

## Conclusion

Whereas LP evidence may ultimately serve to corroborate other evidence, the current lack of consistency in enhancement methods limits practical application of cheiloscopy as a forensic tool in criminal justice delivery. Our review has emphasised the current scope of research regarding physical and chemical enhancement of latent and visible LPs on various substrates, enumerated some limitations, and suggested further research directions to address them. A comprehensive understanding of the structural variations and composition of LPs, as well as a systematic method of development and validation, using robust experimental designs and a more appropriate selection of samples that are reflective of casework situations, will further increase the value of LPs as an additional tool for forensic identification.

## Authors’ contributions

All authors contributed equally to the revision and to drafting the manuscript. All authors contributed to the final text and approved it.
